# Inventory slack routing application in emergency logistics and relief distributions

**DOI:** 10.1371/journal.pone.0198443

**Published:** 2018-06-14

**Authors:** Xianfeng Yang, Wei Hao, Yang Lu

**Affiliations:** 1 Department of Civil and Environmental Engineering, The University of Utah, Salt Lake City, Utah, United States of America; 2 Key Laboratory of Road Traffic Engineering of the Ministry of Education, Changsha University of Science and Technology, Changsha, Hunan, China; 3 Key Laboratory of Safety Design and Reliability Technology for Engineering Vehicle, Changsha University of Science and Technology, Changsha, Hunan, China; 4 Transport & ICT, World Bank, Northwest, Washington DC, United States of America; Beihang University, CHINA

## Abstract

Various natural and manmade disasters during last decades have highlighted the need of further improving on governmental preparedness to emergency events, and a relief supplies distribution problem named Inventory Slack Routing Problem (ISRP) has received increasing attentions. In an ISRP, inventory slack is defined as the duration between reliefs arriving time and estimated inventory stock-out time. Hence, a larger inventory slack could grant more responsive time in facing of various factors (e.g., traffic congestion) that may lead to delivery lateness. In this study, the relief distribution problem is formulated as an optimization model that maximize the minimum slack among all dispensing sites. To efficiently solve this problem, we propose a two-stage approach to tackle the vehicle routing and relief allocation sub-problems. By analyzing the inter-relations between these two sub-problems, a new objective function considering both delivery durations and dispensing rates of demand sites is applied in the first stage to design the vehicle routes. A hierarchical routing approach and a sweep approach are also proposed in this stage. Given the vehicle routing plan, the relief allocation could be easily solved in the second stage. Numerical experiment with a comparison of multi-vehicle Traveling Salesman Problem (TSP) has demonstrated the need of ISRP and the capability of the proposed solution approaches.

## Introduction

Extreme events, both natural and man-made in recent years, including the September 11 attacks (2001), Hurricane Katrina (2005), Sichuan earthquake (2008), Haiti earthquake (2010), and the Fukushima Daiichi nuclear disaster (2011), have highlighted the need of better emergency preparedness and response plans. One critical component of emergency responses is to deliver relief supplies to the impacted population in a timely manner, which could significantly reduce the human suffering and losses. However, as stated in [1], the lack of careful and proactive planning within responsive organizations in the current stage could lead to ineffective allocation and routing strategies, which may worsen the situation in the affected area. Thus, the humanitarian relief logistics problem, which aims at building an integrated and coordinated system for delivering supplies to the right places at the right time and in the right quantities [2], has received increasing research interests in the past two decades.

Although the humanitarian relief logistics problem shares some common features with commercial logistics problems, it also differs from them due to some unique objectives and constraints. For example, in respond to an emergency (e.g., flood, earthquake, hurricanes, terrorist attack, etc.), timely relief supplies, such as blankets, food and medicines, are required to be delivered to the local demand sites (e.g., shelters or other facilities). The operational cost of delivery is no longer the primary concern under this circumstance. Instead, a proper resource allocation and vehicle routing plan is required to ensure in-time delivery to each site, and to prevent the potential running out of relief supplies at any sites. Due to the destabilized infrastructures and uncertainty of the demand in the aftermath of major natural or man-made disasters, the robustness of the allocation and routing plans must be considered.

The importance of above concerns has not been fully recognized by existing studies in the literature until recent years [3–13]. Jabbarzadeh et al. designed a supply chain resilient to major disruptions and supply/demand interruptions. This research conducted a hybrid robust-stochastic optimization model and a lagrangian relaxation solution method. Jabbarzadeh et al. developed dynamic supply chain network design for the supply of blood in disasters. A practical optimization model is developed which can assist in blood facility location and allocation decisions. Bendul and Skorna explored impact factors of shippers’ risk prevention activities. This study will be beneficial for transportation managers to consider the implementation of risk prevention and will support further empirical research and supply chain risk management. Fahimnia et al. developed quantitative models for managing supply chain risks. Cui et al. developed reliable design of an integrated supply chain with expedited shipments under disruption risks. Fattahi et al. developed responsive and resilient supply chain network design under operational and disruption risks. Hasani and Khosrojerdi studied robust global supply chain network design under disruption and uncertainty considering resilience strategies. The model is solved for a real-life case of a global medical device manufacturer to exact managerial insights. Ensafian and Yaghoubi conducted robust optimization model for integrated procurement, production and distribution in supply chain. Govindan et al. studied supply chain network design under uncertainty and this study aims to provide a comprehensive review of studies in this study fields.

Haghani & Oh and Barbarosoglu & Arda’s work [14, 15] still focus on the minimization of the operational monetary cost, where the latter used a finite sample of scenarios as joint realization of uncertain capacity, demand and supply in a two-stage stochastic model. In the studies of Ozdamer et al. and Yi & Ozdamar [16, 17], the objective function has been changed to minimize the unmet demand within the affected region, instead of the operational cost. However, no equity issues were explicitly addressed in their formulation. Sheu treated the headway between two consecutive relief distributions as an important factor to determine the logistics plans [18]. Nolz et al. proposed several meta-heuristics to solve the disaster relief operation problem with the multi-objectives of minimization of tour length and minimization of the latest arrival time of all routes [19]. Pérez, Holguín-Veras et al. discussed the appropriateness of different objective functions used in humanitarian relief logistics problem, and suggested the introduction of deprivation cost from the economics perspective in the decision making process [20–22]. Other studies which are not directly related to the content of this paper include the inventory control at the central distribution depot and the choice of dispensing site locations. For example, Beamon & Kotleba developed an inventory control model that determines the reorder points and order quantities for a long-term emergency relief response [23]. Ukkusuri & Yushimito formulated a facility location problem to preposition relief supplies [24].

The content of this paper is inspired by Herrmann’s work with county public health department for the planning of mass medication distribution to points of dispensing (PODs) from a central location to contend with contagious diseases, and can be viewed as the extension of [25]. Herrmann et al. [26] compared the emergency relief supplies planning problem with the conventional vehicle routing problem (VRP) [27, 28] and inventory routing problem (IRP) [29]. They argued that a slack, which is defined as the difference between the time new deliveries arrive and that when all previously delivered inventory will be exhausted, is an appropriate measure for the robustness of the distribution plan under network and demand uncertainties. They introduced a new problem named Inventory Slack Routing Problem (ISRP) to maximize the minimum slack over all the dispensing sites, thus minimizing the probability of running out of relief supplies. To develop an efficient solution algorithm, Herrmann et al. later proposed a two-stage approach for the Planning Medication Distribution (PMD) problem [30]. The problem was separated into a routing problem that creates routes for each delivery vehicle and a scheduling problem that determines when and how much medication should be delivered to each site. To further improve the solution algorithm, Montjoy & Herrmann integrated the adaptive large neighborhood search method into previously proposed models to deal with ISRP [31, 32]. After exploring the relationship between the objective function and decision variables, Yang & Feng proposed efficient solution heuristics to solve the ISRP [25]. In the routing stage, they sorted the sites with their dispensing rate and the one with the largest dispensing rate was given the highest priority when deciding the routing plan. The sites were then inserted to different routes according to their priorities, and the route which could minimize the latest arrival time was chosen for that specific site.

In Herrmann’s setting, the number of vehicles is relatively large compared with the number of sites. For example, they used 25 vehicles serve a network with 50 sites. The heuristic in [25] also work sufficiently well under this situation. However, when the number of vehicles is no longer a loose constraint (for instance, 6 vehicles for 50 sites), if the sites are inserted strictly based on their priorities, the vehicle may need to visit two sites which are far away from each other consecutively, which is definitely not the optimal plan. In this paper, two improved heuristics were proposed to tackle this problem in the routing stage. In the first heuristic, the priority is not strictly enforced and different levels of relaxation are introduced during the insertion process [33]. In the second heuristic, the basic idea of sweep algorithm [34] is incorporated to impose restrictions on the area one vehicle could travel. In the scheduling stage, equal slack is ensured for all the sites based on their dispensing rate. The results on a hypothetical network showed clear improvement on the resulted minimum slack.

## Inventory slack routing problem

### Problem description

**Fig 1:**
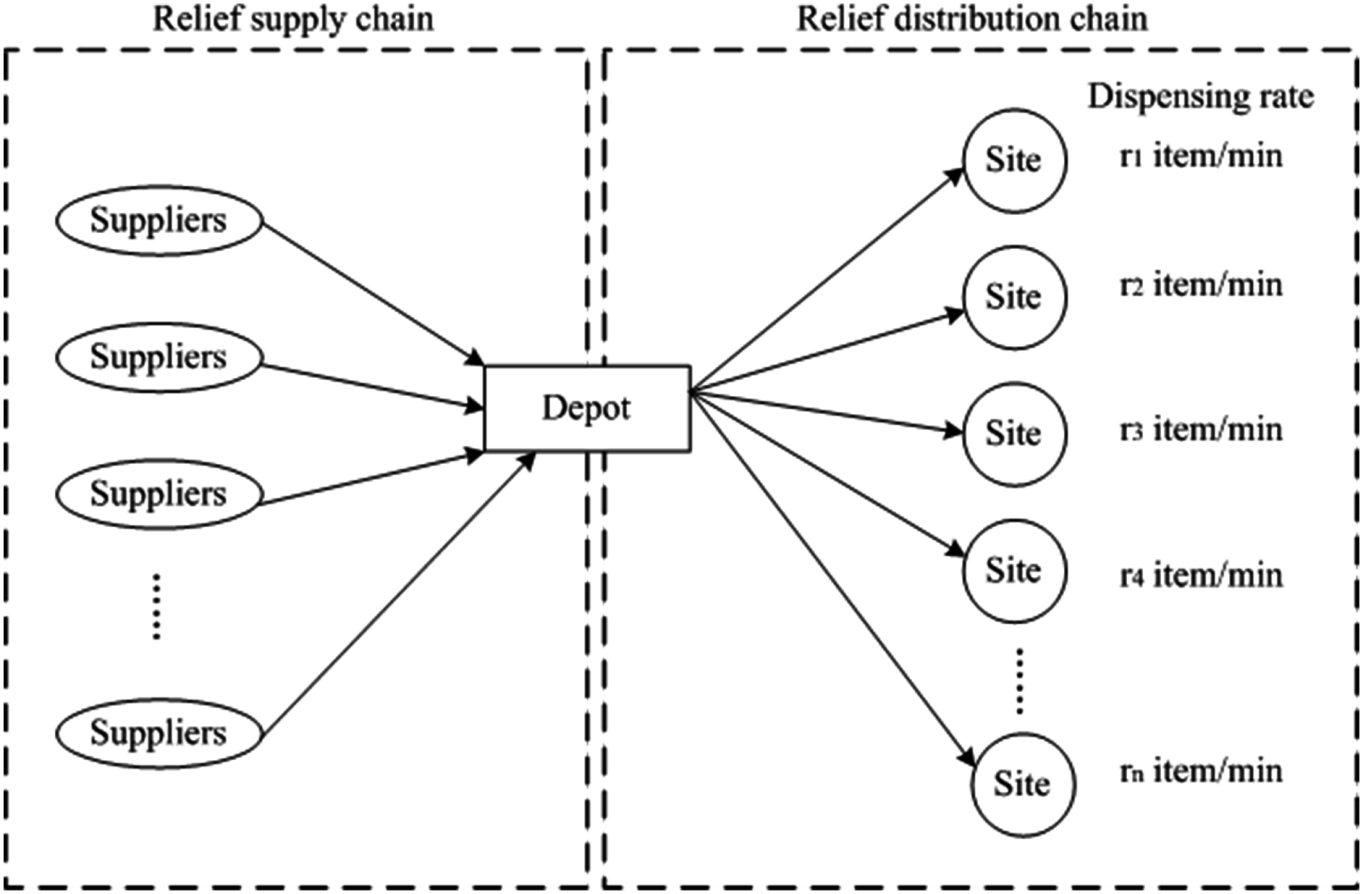
The relief logistic network.

In the aftermath of natural or manmade disasters, such as an earthquake, tsunami, or hurricane, there are urgent demands at many locations for relief supplies. Hence, to reduce the impact caused by the disasters, those relief supplies shall be distributed to the public as soon as possible. In response to such need, the hypothetical relief logistic network depicted in Fig 1 involves three primary chain members: relief suppliers; distribution depot; and dispensing sites. Table 1 shows the notions for the inventory slack routing problem.

**Table 1:** Notions for the inventory slack routing problem.

Variable	Description
*I*_*k*_	Total amount of reliefs delivered to the depot in batch *k*;
*Ω*	The set of demand sites with a size of *N*;
*Ψ*	The set of delivery batches with a size of *U*;
*M*	Vehicle fleet size at the depot;
*q*_*ik*_	The amount of reliefs delivered to site *i* in batch *k*;
*Q*_*ik*_	The total amount of reliefs delivered to site *i* before batch *k*;
*L*_*i*_	The dispensing rate of site *i*;
*R*_*i*_	The starting inventory level at site *i*;
*c*_*ij*_	The travel time between site *i* and *j*;
*p*	The vehicle unloading time at each site;
*t*_*ik*_	The arriving time of reliefs at site *i* in batch *k*;
*T*_*k*_	The arriving time of reliefs at the depot from suppliers in batch k;
*w*_*i*_	The time duration between *t*_*ik*_ and *T*_*k*_;
*x*_*ij*_	The routing decision variable (equals to “1” if site *i*, *j* are connected and *i* is visited before *j*);
*S*_*ik*_	The slack of site *i* in batch *k*;

Based on the hypothetical relief logistic network, two vital issues could be identified here: the management of relief supply chain and the vehicle routing design for relief distributions. In this study, we assume the arriving time of relief supplies at the depot is given and only focus on the relief distribution issue. Also, to facilitate the modeling and discussion, several assumptions are introduced for system operations: i) at the relief supply layer, the reliefs are delivered to the depot (with different quantities) in several “batches” at time *T*_*1*_, *T*_*2*_, … *T*_*M*_, respectively; ii) each demand site has a specified dispensing rate and will be served by only one vehicle; iii) each single vehicle has enough capacity to satisfy the needs of demand. Hence, two crucial problems are raised in the relief distribution process: vehicle routing design and relief allocation.

**Fig 2:**
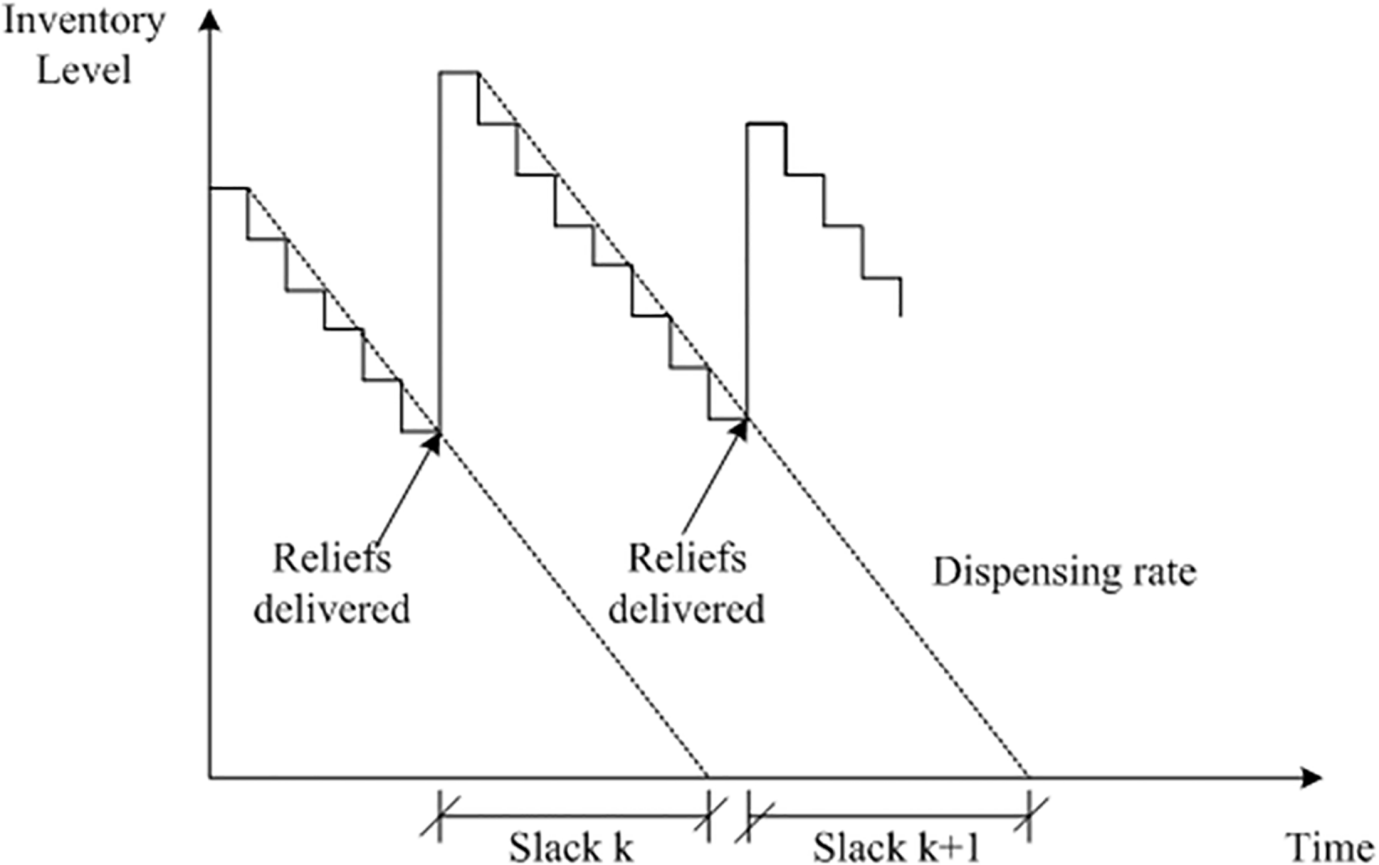
Illustration of “Slack” at demand sites.

Given the starting inventory levels and the dispensing rates of demand sites, one can recognize that those having lower inventory level and larger dispensing rate are in more urgent need of relief supplies. To dealing with this situation, Panchamgam et al. proposed a hierarchical traveling salesman problem (HTSP) by assigning each demand site with a specified priority level [33]. Then the vehicle must visit the site in an order that strictly respect to the priority levels. The inventory slack routing problem (ISRP) has the similar objective but with several additional concerns. As shown in Fig 2, the slack at demand sites is defined as the duration between the reliefs arriving time and the estimated inventory stock-out time. Hence, a negative value of slack will represent the occurrence of stock-out. The control objective of ISRP is to maximize the slacks at demand sites so as to face the potential delivery lateness due to the uncertainty of vehicle travel time in practice.

Given the travel time between demand sites and central distribution depot, one can obtain the reliefs arriving at site *i* in batch *k* of delivery using the following equations:

$$t_{i,\text{k}}=T_{k}+w_{i}$$ (1)

$$w_{i}=\sum_{j\in \Omega }^{}(x_{ji}w_{j}+c_{ji}+p)$$ (2)

Hence, the slack of site *i* in batch *k* of delivery can be derived by:

$$Q_{ik}=\sum_{j=1}^{k-1}q_{ij}$$ (3)

$$S_{i,k}=\frac{R_{i}+Q_{ik}}{L_{i}}-t_{i,k}$$ (4)

Regardless of the different dispensing rate at demand sites, the occurrence of stock-out at any locations may cause significant impact to the disaster suffers. Therefore, the ISRP provides an objective function that maximizes the minimal slack among all the sites in each batch of delivery:

$$Max\{\underset{i\in \Omega }{\text{min}}(S_{i,k})\}$$ (5)

### Problem decomposition

To efficiently find the solution, the inventory slack routing problem defined above can be decomposed as two inter-related sub-problems: vehicle routing and reliefs allocation.

#### Vehicle routing

**Fig 3:**
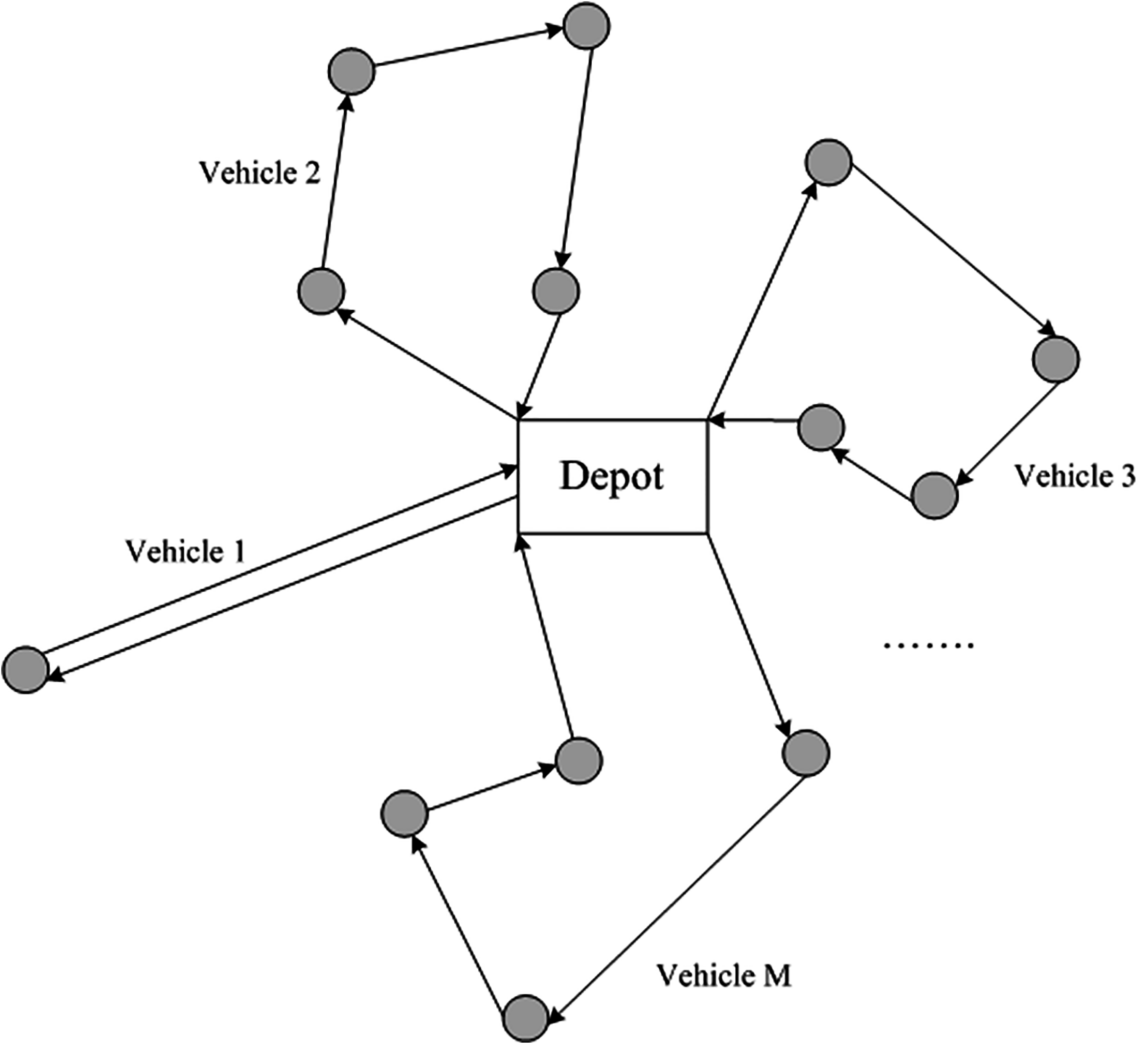
Illustration of the routing sub-problem.

Based on the travel time between the central depot and demand sites, the vehicle routing sub-problem is to design the routing plan for all the available vehicles that can visit each demand site. Fig 3 shows an illustrative example of this sub-problem. Given the vehicle fleet size *M*, three core issues shall be accounted in this sub-problem: 1) how to balance the workload of vehicles to achieve an optimal solution; 2) what kind of objective function is suitable for the routing design; 3) how to design the delivery sequence of each vehicle.

As shown in Eq (4), the objective of maximizing minimal slack is determined by both routing decision variables and reliefs assignment quantities. Hence, to design an optimal routing plan, one shall firstly analyze the inter-relationship between these two sub-problems.

#### Relief allocation

**Fig 4:**
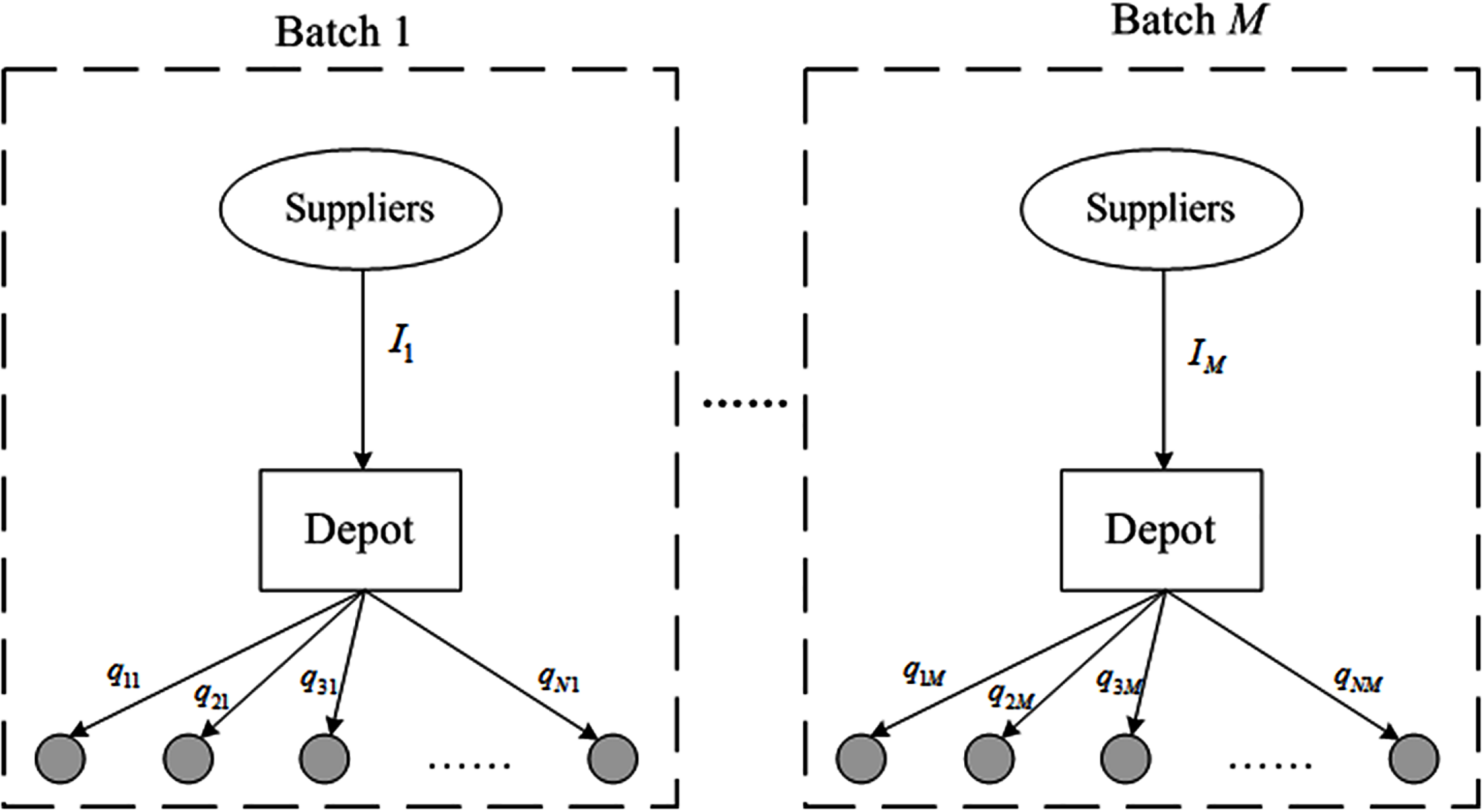
Illustration of relief allocation problem.

In response to the different dispensing rates at those demand sites, the relief allocation problem is to determine the quantities of reliefs assigned to the sites in each delivery batch, based on the obtained vehicle routing plans. The typical process is shown in Fig 4.

Given the vehicle routings, the relief allocation sub-problem can be described as a LP model:

MaxS (6)

$$\begin{array}{lll} s.t. & & \\ & \sum_{i\in \Omega }q_{ik}=I_{k} & \forall k \end{array}$$ (7)

$$\;t_{i,\text{k}}=T_{k}+w_{i}\;\;\;\;\forall k$$ (8)

$$Q_{ik}=\sum_{j=1}^{k-1}q_{ij}\;\;\;\;\;\forall k$$ (9)

$$\;S\leq \frac{R_{i}+Q_{ik}}{L_{i}}-t_{ik}\;\;\;\;\forall k$$ (10)

$$q_{ik}\geq 0\;\;\;\;\;\;\;\;\;\;\;\;\;\;\;\;\;\forall i,k$$ (11)

As shown in [30], an optimal solution to such a LP will ensure every site has the identical slack in each delivery batch.

## Two-stage solution approach

In response to the two sub-problems defined above, the solution algorithm includes two sequential stages.

### Stage1: Vehicle routing design

#### Approach fundamentals

To establish a solution heuristic for the vehicle routing problem in ISRP, the first task is to explore a proper objective function. Based on the assumption that the allocation of reliefs will ensure every site has identical slack in each delivery batch, one can re-write Eq (4) as:

$$S_{i,k}=\frac{R_{i}+Q_{ik}}{L_{i}}-(w_{i}+T_{k})=S_{k}$$ (12)

Then,

$$R_{i}+Q_{ik}-w_{i}L_{i}-T_{k}L_{i}=S_{k}L_{i}$$ (13)

Summarize the both side of Eq (13) with respect to *i*:

$$\sum_{i\in \Omega }^{}R_{i}+\sum_{i\in \Omega }^{}Q_{ik}-\sum_{i\in \Omega }^{}w_{i}L_{i}-T_{k}\sum_{i\in \Omega }^{}L_{i}=S_{k}\sum_{i\in \Omega }^{}L_{i}$$ (14)

Therefore:

$$S_{k}=\frac{\sum_{i\in \Omega }^{}R_{i}+\sum_{i\in \Omega }^{}Q_{ik}-\sum_{i\in \Omega }^{}w_{i}L_{i}-T_{k}\sum_{i\in \Omega }^{}L_{i}}{\sum_{i\in \Omega }^{N}L_{i}}$$ (15)

Eliminate the known parameters in Eq (15), the problem of maximizing slack can be converted to:

$$\text{min}\;\;\sum_{i\in \Omega }^{}w_{i}L_{i}$$ (16)

Hence, one can use Eq (16) to be the objective function in the vehicle routing sub-problem.

#### Hierarchical routing approach

According to the objective function shown in Eq (16), it should be recognized that a better solution might be achieved if those sites with larger dispensing rates can receive shorter delivery duration *w*_*i*_. In other words, those high rate sites shall obtain the delivery priority when designing the vehicle routings. To satisfy such need, a hierarchical routing approach with a relaxation degree of *r* is introduced as follows:

- Step 1: Sort those demand sites in a descending order *H* according to their dispensing rates. Let the depot be the first site in each vehicle route *j*.
- Step 2: Select the prior *r* sites in the list *H* and denote them as a set ζ; for each site *i* in *ζ*, compute its minimum *w*_*i*_ by the following equation:
  $$w_{i,\text{min}}=\underset{j=1,\ldots ,M}{\text{min}}\{w_{l,j}+c_{li}+p\}$$ (17)
  where, *l* in *w*_*l*,*j*_ is the index of the last site in route *j*.
  Compare the *w*_*i*,*min*_ of these *r* sites in *ζ*, select the one with the smallest *w*_*i*,*min*_ to be the target site; insert the target site into its optimal route (which provides *w*_*i*,*min*_) and remove it from list *H*;
- Step 3: Denote the size of *H as |H|*. If *|H|> = r*, go to Step 2; if *|H| = 0*, *stop; if 0<|H|<r*, then let *r* = *|H|* and go back to Step 2.

**Fig 5:**
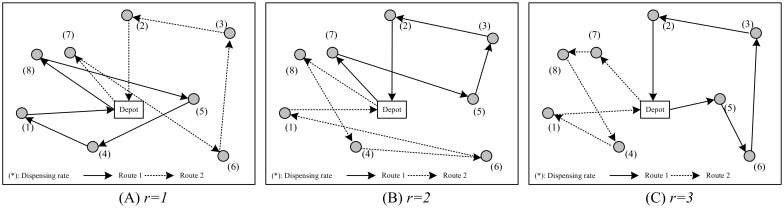
An illustrative example for the hierarchical routing problem.

When the *r* equals to “1”, the hierarchical routing approach will be identical to the heuristic provided in [25]. As shown in Fig 5(A), in a worst case condition, the hierarchical approach may produce an extremely long route length. With the increasing of relaxation parameter *r*, the total routing distance can be decreased, as shown in Fig 5(B) and 5(C). In view of objective function shown in Eq (16), the reduction of total routing distance might decrease the average delivery duration {*w*_*i*_} and consequently improve the routing solution. However, since those “urgent” sites have the largest dispensing rate {*L*_*i*_}, an optimal routing plan may intend to offer delivery priority to those sites. In most cases, there is inevitably a tradeoff between the efficiency and emergence, and the selection of relaxation parameter *r* will become a vital issue in this heuristic. At the current stage, no related theoretical analysis is available and different value of *r* will be tested in the computation experiment section.

#### A sweep approach

As shown in the converted objective function shown in Eq (16), the routing design in ISRP shall account for both delivery duration and dispensing rate of demand sites. Also, designing routings with multi-vehicles has increased the computation complexity. To satisfy such need, the sweep approach needs to account for two primary issues: 1) to determine the delivery priority of each site according to its dispensing rate and distance to the depot; 2) to balance the workload of vehicles.

In response to the first issue, this study defines gravities between pairs of sites and depot:

$$G_{ij}=\left\{ \begin{array}{l} L_{i}L_{j}/c_{ij}{}^{\alpha }\text{if}\;\text{i,}\;\text{j}\;\text{are}\;\text{dispensing}\;\text{sites}\\ L_{i}I_{j}/c_{ij}{}^{\alpha }\text{if}\;\text{i}\;\text{is}\;\text{dispensing}\;\text{site}\;\text{and}\;\text{j}\;\text{is}\;\text{depot} \end{array}\right.$$ (18)

where, *α* is a parameter.

To address the second issue, the sweep approach divides the entire service area into *M* sub-areas and each of them will be served by one vehicle. For the depot indexed by *N+1*, we use the sum of *L*_*i*_*c*_*N+1*,*i*_ within each sub-area to represent the workload of the serving vehicle. Hence, the division of service area needs to minimize the variance of vehicles’ workload.

**Fig 6:**
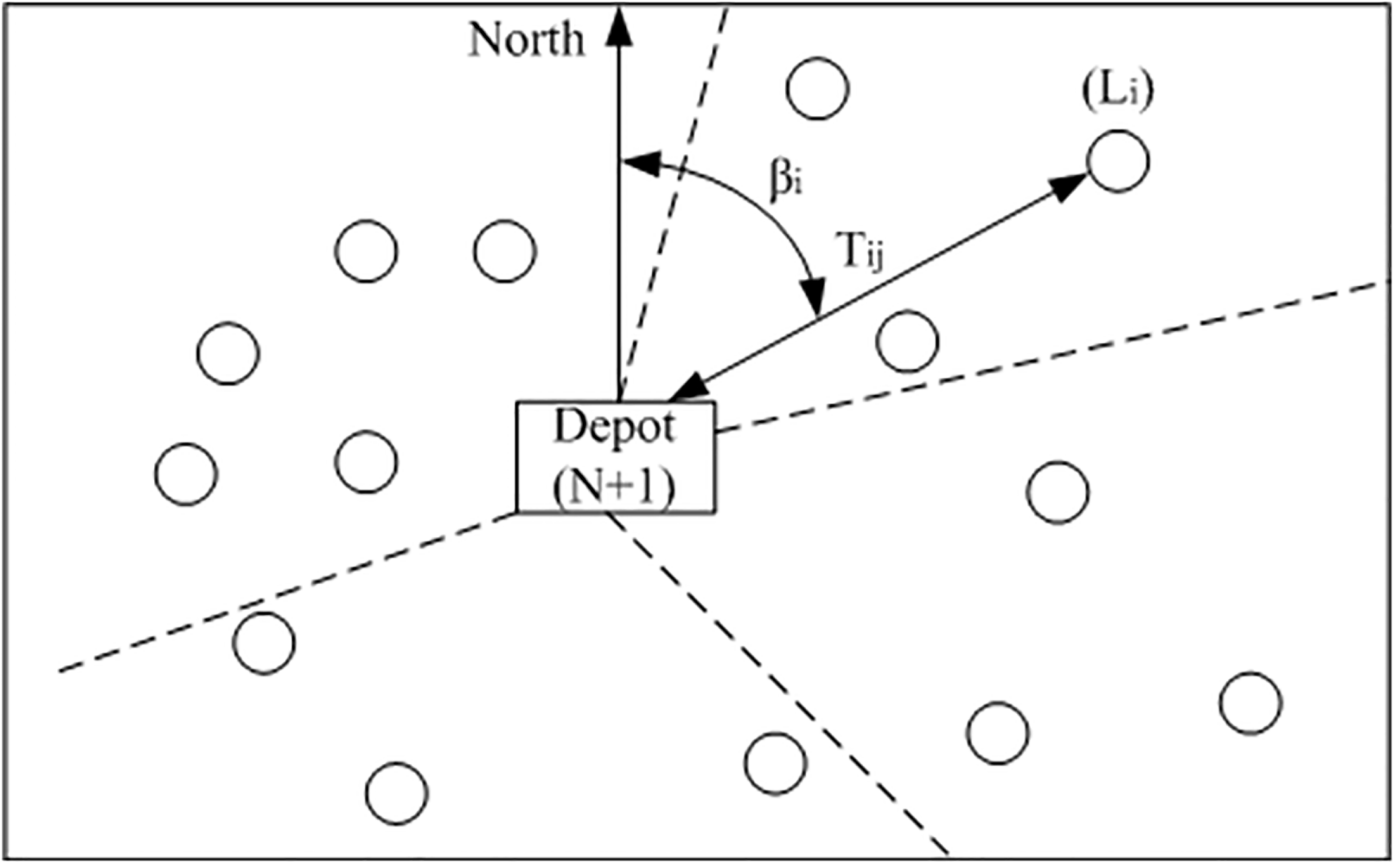
The separation of sub-areas for individual vehicles.

Using the nations defined in Fig 6, the sweep approach could be given as follows:

- Step 0: compute the *L*_*i*_*c*_*N+1*,*i*_ of each demand sites *i*; then the average workload of each vehicle could be obtained by ∑ *L*_*i*_*c*_*N+1*,*i*_ */M*;
- Step 1: sort the demand sites as an ascending list based on the angle of *β*_*i*_;
- Step 2: select one site in the ascending list as the beginning, then continue to sweep its following sites and stop when the total *L*_*i*_*c*_*N+1*,*i*_ of swept sites exceeds ∑ *L*_*i*_*c*_*ij*_ */M*; the swept area will be identified as one sub-area;
- Step 3: at the stopped site obtained in Step 2, repeat the process until all the sites have been swept and *M* sub-area are obtained; compute the resulting variance of vehicle’s’ workload among the *M* sub-areas;
- Step 4: select another site as the beginning and repeat Step 2-Step3 until an optimal plan is found.
- Step 5: for each sub-area, the vehicle will firstly serve the site with largest gravity to the depot, and move to another site having the largest gravity to the last site in route. Stop when all the sites are connected.

## Stage 2: Relief allocation problem

To solve the relief allocation problem, one can directly solve the LP model stated in Eqs (6)–(11). However, a much easier method can be derived by taking the conclusion that all the demand sites will have identical slack at optimality.

Based on the definition of slack in Eq (4), one can substitute *S*_*i*,*k+1*_ by *S*_*i*,*k*_ and get:

$$S_{i,k+1}=\frac{S_{i,k}L_{i}+q_{i,k}}{L_{i}}-[(T_{k+1}+w_{i})-(T_{k}+w_{i})]=S_{i,k}+\frac{q_{i,k}}{L_{i}}-(T_{k+1}-T_{k})$$ (19)

Summarize all the sites served by the target depot *m*, one can get:

$$\sum_{i\in \Omega }L_{i}S_{i,k+1}=\sum_{i\in \Omega }\left[S_{i,k}L_{i}+q_{i,k}-(T_{k+1}-T_{k}\right)L_{i}]$$ (20)

Using *S*_*k*_ to represent the identical slack in delivery batch *k*:

$$S_{k+1}\sum_{i\in \Omega }L_{i}=\sum_{i\in \Omega }S_{i,k}L_{i}+\sum_{i\in \Omega }q_{i,k}-\sum_{i\in \Omega }\left[(T_{k+1}-T_{k}\right)L_{i}]$$ (21)

Then, the slack could be computed by:

$$S_{k+1}=\frac{\sum_{i\in \Omega }S_{i,k}L_{i}+I_{k}-\sum_{i\in \Omega }\left[(T_{k+1}-T_{k}\right)L_{i}]}{\sum_{i\in \Omega }L_{i}}$$ (22)

Then, Eq (19) could be re-written as:

$$q_{i,k}=(\frac{\sum_{i\in \Omega }S_{i,k}L_{i}+I_{k}-\sum_{i\in \Omega }\left[(T_{k+1}-T_{k}\right)L_{i}]}{\sum_{i\in \Omega }L_{i}}-S_{i,k}+T_{k+1}-T_{k})L_{i}$$ (23)

It should be noted that one important assumption to guarantee the achievement of identical slack of each site is the sufficient capacity of vehicles.

### Solution upper-bound

To evaluate the effectiveness of the proposed heuristic, a solution upper-bound shall be explored for comparison. Montjoy and Herrmann introduced a method that assumes each site can be served by one individual vehicle [31]. The solution upper-bound obtained by this method is tight enough when the number of vehicles is sufficient (e.g, 25 vehicles for 50 sites in [25]). However, with the decreasing of number of vehicles, a tighter bound will be required.

Given the number of demand sites *N* and the number of vehicles *M*, one can observe a fact that: At most *M* sites can be the *n*th site in the routes and the rest *N* − *nM* sites will at least be the (*n*+1)th sites in the routes, where *n* is a integer less than *N*/*M*. For the convenience of analysis, let’s define *T*_*i*,*n*_ as the minimum delivery duration to site *i* if it is the *n*th sites in the route. Hence,

$$T_{i,n}=\{\begin{array}{ll} c_{N+1,i}+p & if\;n=1\\ \underset{\forall r_{j}\in \Omega }{\text{min}}\{c_{N+1,r_{1}}+\sum_{j=1}^{n-2}c_{r_{j},r_{j+1}}+c_{r_{n-1},i}\}+np & if\;n>1 \end{array}$$ (24)

Hence, the computation complexity of *T*_*i*,*n*_ is *O*(*N*^*n*^). We also define:

$$\Delta_{i,n}=T_{i,n}-T_{i,n-1}\;for\;n\neq 1$$ (25)

Then, one solution lower-bound for the vehicle routing problem based on Eq (16) could be obtained by:

$$\sum_{i\in \Omega }^{}w_{i}L_{i}\geq \sum_{i\in \Omega }T_{i,n}L_{i}-\sum_{j=2}^{n}\sum_{i\in \sigma_{j}}\Delta_{i,j}L_{i}$$ (26)

Here *σ*_*j*_ is a subset of Ω and it contains *M* sites having the largest Δ_*i*,*j*_*L*_*i*_.

Obviously, increasing the parameter value of *n* will requires more computation time but the lower-bound of vehicle routing sub-problem will become tighter.

Then one can easily compute the upper-bound of the ISRP by Eq (27):

$${\tilde{S}}_{k}=\frac{\sum_{i\in \Omega }^{}R_{i}+\sum_{i\in \Omega }^{}Q_{ik}-(\sum_{i\in \Omega }T_{i,n}-\sum_{j=2}^{n}\sum_{i\in \sigma_{j}}\Delta_{i,j})-T_{k}\sum_{i\in \Omega }^{}L_{i}}{\sum_{i\in \Omega }^{}L_{i}}$$ (27)

## Computational experiment

The developed two-stage solution approach for the Inventory Slack Routing Problem is coded in Matlab. The computational experiments are conducted on a PC with 3 GHz CPU and 8GB RAM running Windows 7 operating system.

### Experimental design

A network consists of 1 depot and 50 demand sites is employed to test the performance of the proposed two-stage approach. The vehicle fleet size at the depot is 6 and the unloading time at each site is 10 minutes. Reliefs from the suppliers would arrive at the depot in three batches, and the starting time of each delivery batch and relief quantities to be distributed are given by Table 2.

**Table 2:** Delivery quantity arriving at depot in each batch.

Delivery Batches	Starting time (mins)	Reliefs Quantity (units)
Batch 1	100	150000
Batch 2	700	170000
Batch 3	1100	190000

Also, the dispensing rate (DR) and starting inventory level (SIL) at each site are given in Table 3:

**Table 3:** List of dispensing rates and starting inventory levels.

Site	DR	SIL	Site	DR	SIL	Site	DR	SIL	Site	DR	SIL	Site	DR	SIL
*1*	5	2500	*11*	14	7000	*21*	15	7500	*31*	9	4500	*41*	11	5500
*2*	5	2500	*12*	11	5500	*22*	12	6000	*32*	3	1500	*42*	14	7000
*3*	6	3000	*13*	8	4000	*23*	8	4000	*33*	5	2500	*43*	13	6500
*4*	1	500	*14*	11	5500	*24*	8	4000	*34*	10	5000	*44*	15	7500
*5*	4	2000	*15*	2	1000	*25*	10	5000	*35*	11	5500	*45*	1	500
*6*	5	2500	*16*	10	5000	*26*	7	3500	*36*	5	2500	*46*	3	1500
*7*	13	6500	*17*	14	7000	*27*	9	4500	*37*	2	1000	*47*	12	6000
*8*	2	1000	*18*	8	4000	*28*	12	6000	*38*	12	6000	*48*	1	500
*9*	4	2000	*19*	4	2000	*29*	5	2500	*39*	14	7000	*49*	10	5000
*10*	14	7000	*20*	8	4000	*30*	14	7000	*40*	7	3500	*50*	3	1500

### Sensitivity analysis

**Fig 7:**
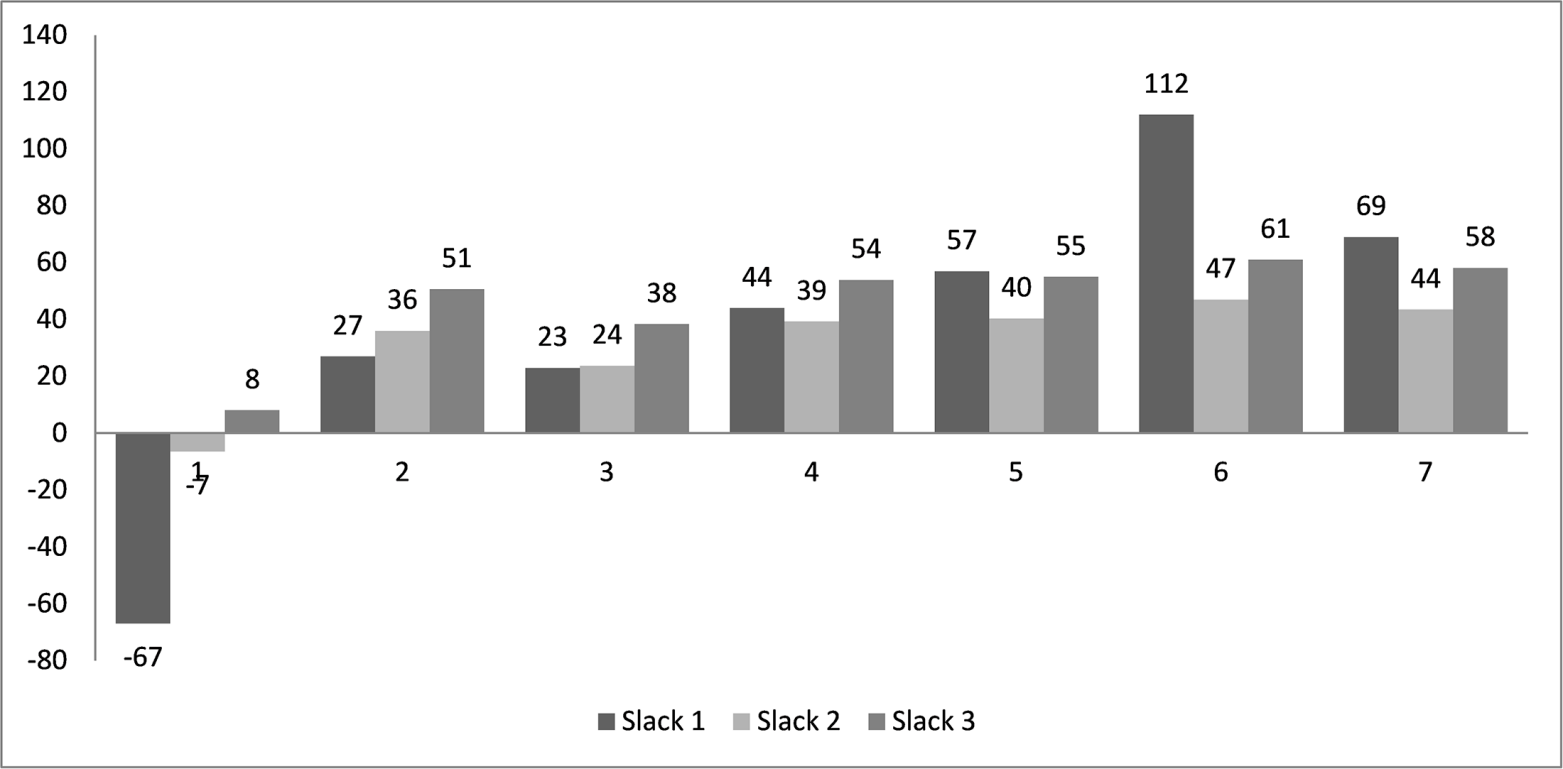
Sensitivity analysis of the relaxation parameter *r*.

As stated in Fig 5, the selection of relaxation parameter *r* in the hierarchical routing approach can affect the optimality of solution. In this section, a sensitivity analysis is conducted to calibrate this parameter. Fig 7 reports the minimum slack within each batch under various parameter values for the given network. It is observed that two slacks are negative when *r* equals 1. With the increasing of *r*, the slacks become larger. One possible reason is that the relaxation of delivery priority can help to allocate the sites to a proper route so as to reduce the delivery durations. The optimal objective value could be found when r equals 6 since an over-relaxed routing plan will delay the arriving time of relief at those urgent sites.

### Computational results

**Fig 8:**
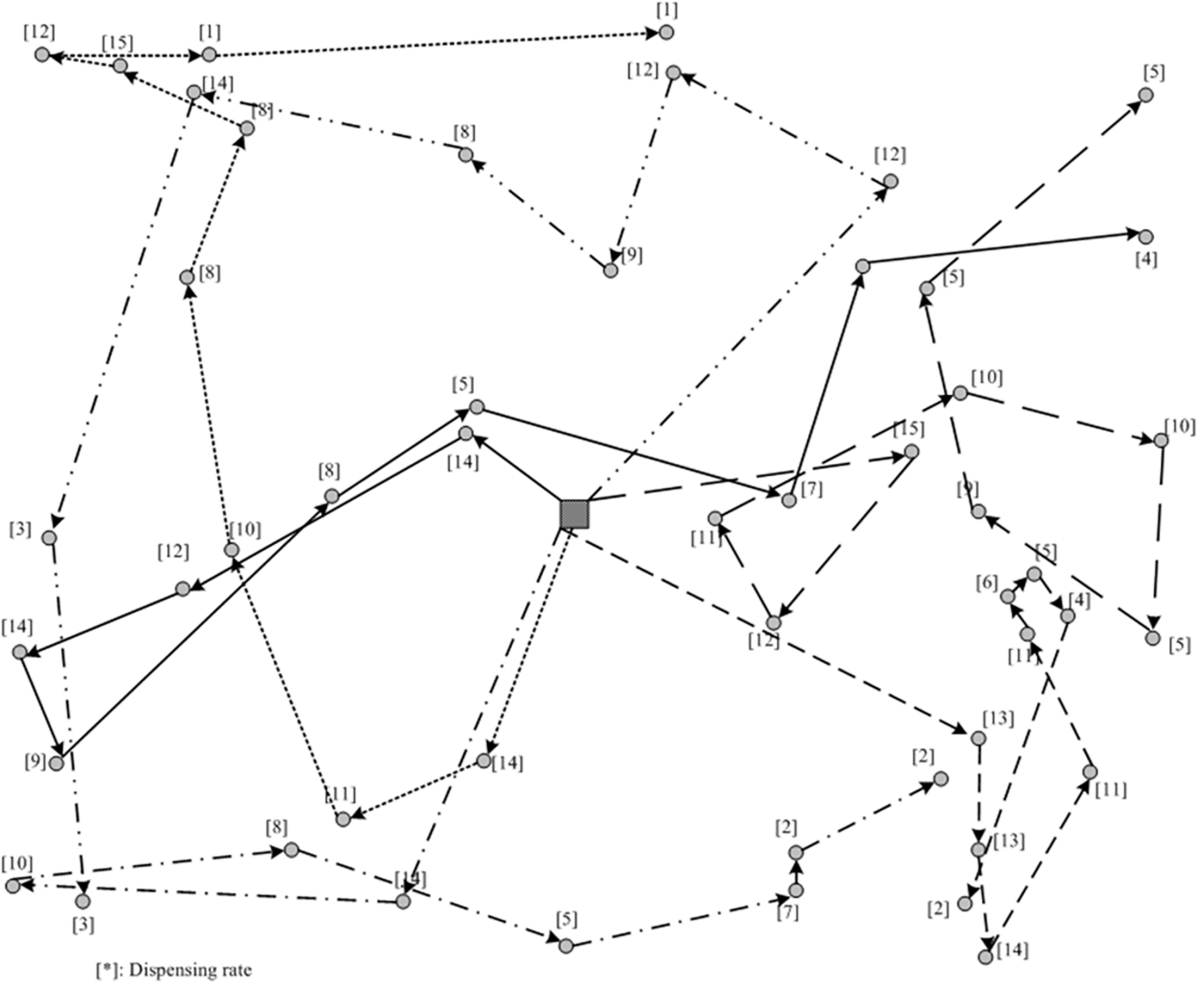
Resulted vehicle routing plan by the hierarchical routing approach.

Based on the sensitivity analysis, we select the value of parameter *r* to be 6, and the corresponding routing plan is shown in Fig 8. Note that the returning line from the last site to the depot in each route has been eliminated in the figure. By examining the obtained vehicle routing plans, one can observe that those urgent sites with larger dispensing rates can receive the delivery priority in their routes. However, to satisfy the hierarchical requirement, a lot of route crossovers can be observed and the total travel time will inevitably become significant larger compared with a traditional TSP solution.

**Fig 9:**
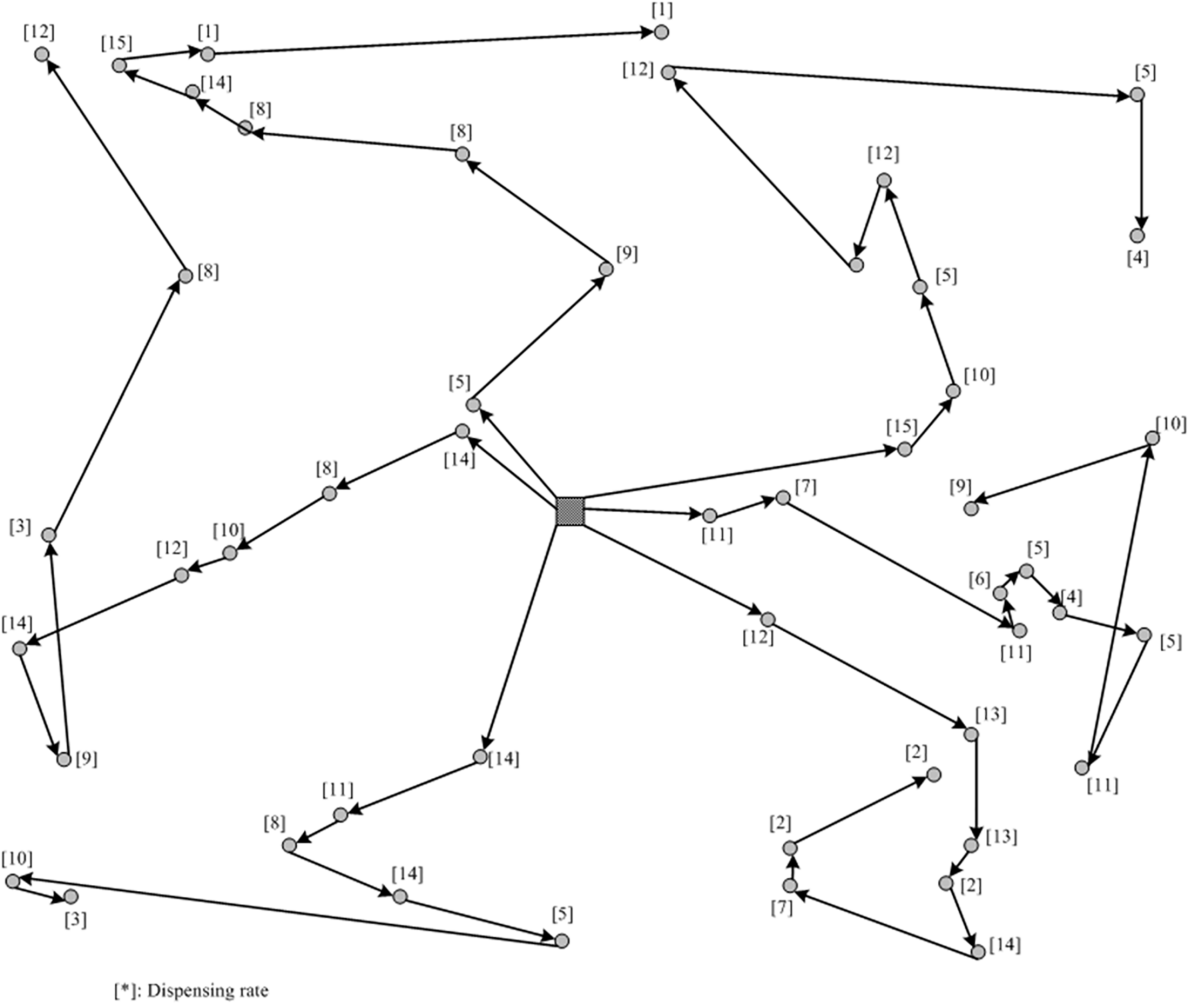
Resulted vehicle routing plan by the sweep approach.

As shown in Fig 9, the sweep approach has divided the entire network into 6 sub-areas and each vehicle will serve one particular sub-area. Since the delivery sequence by each vehicle is determined by the gravities which determined by both dispensing rates and distances between sites, those urgent sites will not always receive the delivery priority within their routes. Obviously, the sweep approach can produce a routing plan with much smaller total travel time compared with the hierarchical routing approach.

Given the routing plans, one can determine the relief allocation and compute the resulting slack in each delivery batch using Eq (23).

To illustrate the need of ISRP, a solution obtained by traditional multi-vehicle TSP is also employed for comparison. Assuming all the demand sites have an identical dispensing rate, the sweep approach introduced in this study can be used to divide sub-areas for the multi-vehicle TSP. Then the Christofides algorithm is used to design the routing in each sub-area. The computation results from these three approaches are compared. Also, several MOEs are interested in this study, including the three minimal slacks, computation time and total travel time. The upper-bound of slacks are computed with *n* = 3. Table 4 summarizes the computational results for these three approaches.

**Table 4:** Computational results of different approaches.

	Hierarchical routing approach	Sweep approach	Multi-vehicle TSP
Computation Time (sec)	<0.1	28	28
Minimum Slack 1 (min)	112	165	32
Minimum Slack 2 (min)	47	68	17
Minimum Slack 3 (min)	61	83	24
Total travel time	1093	765	678
Slack 1/upper-bound	0.566	0.833	0.161
Slack 2/upper-bound	0.580	0.839	0.210
Slack 3/upper-bound	0.604	0.822	0.237

Obviously, the efficiency of routing plan at the first stage can significant affect the resulting slacks. By ignoring the difference of dispensing rate, the approach for multi-vehicle TSP has generated the smallest slacks even though its travel time is shortest. Hence, one can observe the difference between ISRP and TSP and recognize the importance of accounting dispensing rate in the vehicle routing design stage. Comparing the two proposed approaches, the hierarchical routing approach can require a much shorter computation time. However, regarding to the minimum slacks, the sweep approach can produce larger slacks and shorter travel time. Therefore, the sweep approach can outperform the hierarchical routing approach in this computational experiment. By analyzing the fundamentals of the two proposed approaches, the hierarchical routing approach devotes more efforts on treating dispensing rates while the sweep approach defines gravities to balance the impact of both dispensing rate and distance to the depot of sites, which might be a reason to explain the better performance of the sweep approach.

## Conclusions

To conclude, this study has proposed a two-stage solution approach for the Inventory Slack Routing Problem (ISRP) with consideration of two decision sub-problems: vehicle routing and relief allocation. By analyzing the inter-relations between these two sub-problems, the objective of maximizing the minimal slack was converted into a new objective consists of both delivery duration and dispensing rate of demand sites. Based on the converted objective function, two solution approaches, hierarchical routing approach and sweep approach, were proposed at the first stage. Then a set of equations were derived to solve the relief allocation problem at the second stage. The developed approaches have been tested by an experimental network and have shown the capability of producing high quality solutions with a reasonable time. Also, with the comparison of total travel time and resulting minimal slacks, the sweep approach can outperform the hierarchical routing approach in this case.
